# The Internal and External Action of Thymic Acid and Thymate of Soda

**Published:** 1879-10

**Authors:** 


### THE INTERNAL AND EXTERNAL ACTION OF THYMIC ACID AND THYMATE OF SODA.

Dr. Alvin, quoted in the *Practitioner*, from *Le Progres Medicale*, has for more than a year substituted thymic for carbolic acid, in preparations which are used to produce a caustic, substitutive, or simply astringent action upon the mucous membranes of the larynx and pharynx. The solutions thus prepared are much better borne, and are at the same time much more active than those at present in use, and have given the most satisfactory therapeutic results. Thymic acid has the same curative properties as carbolic acid, and has a more pleasant smell. Some of the formulæ which extended use upon a large number of patients has shown to be capable of giving good results are here appended. Solutions for painting the parts may be made as follows:—

1. (*a*) Crystallized thymic acid, one; pure glycerin, two to four; (*b*) crystallized thymic acid, one; iodine, one; potassium iodide, one or two; pure glycerin, five to fifteen.

2. Substitution solutions: (*c*) crystallized thymic acid, one; pure glycerin, fifty; (*d*) crystallized thymic acid, one; iodine, one; potassium iodide, one to two; pure glycerin, one hundred and twenty; (*e*) crystallized thymic acid, one; tannin, one; pure glycerin, one hundred.

3. (*f*) Astringent solutions; crystallized thymic acid, one; pure glycerin, fifty.

4. (*a*) Pastils: thymate of soda, one milligram. To make a pastil of one gram.

For superficial stomatitis; irritation of the tract; softening of the mucous membrane in smokers; they are very efficacious in cases of spasmodic cough. They have also been tried in whooping cough.

(*b*) Thymate of soda, one milligram; chlorate of potash, ten centigrams. For a pastil of one gram.

In severe stomatitis, tonsillitis, and pharyngo-laryngitis.

(*c*) Thymate of soda, one milligram; borax, ten centigrams. For a pastil of one gram.

Ulcerated stomatitis and tonsillitis.

The pastils should be taken in doses of six to ten a day. They are calculated to remove the putridity of the parts which they are destined to cure.

5. Drink: thymate of soda, one to four centigrams; simple syrup, sixty-one grams; water, one hundred grams. To be taken in twenty-four hours.

This preparation has been invariably used with success in affections of the lungs, the cough being lessened and the expectoration modified.

In cases of catarrhal bronchitis the disease has always been lessened in duration, and has been occasionally abated. Without stating that it is efficacious in whooping cough, Dr. Alvin believes that it will be found to be useful in that disease, judging from the effects produced on the few patients upon whom he has tried the remedy. Thymic acid has also been employed for inhalation, and in aqueous solution for cases in which naso-pharyngeal irrigation was indicated. It is also recommended as an exceedingly valuable modificatory agent. Thymic acid often contains impurities, and it is to obviate this imperfection that the crystallized acid is ordered. Thymate of soda is a very unstable salt, and requires great care in its preparation. Thymic acid, which is a powerful caustic, has hitherto been prescribed in too large doses (one gram to one hundred and fifty grams in twenty-four hours). Thymic acid was used in 1866, by Giraldes, in the treatment of wounds.

